# Multi-method analysis of gender differences in psychological distress among the elderly during COVID

**DOI:** 10.1093/eurpub/ckac130.207

**Published:** 2022-10-25

**Authors:** C Queen, R Pasupathy, I Reine

**Affiliations:** 1 Department of Public Health, Texas Tech University Health Sciences Center, Abilene, USA; 2 Institute of Public Health, Riga Stradins University, Riga, Latvia; 3 Department of Public Health and Caring Sciences, Uppsala University, Uppsala, Sweden

## Abstract

As COVID swept through Europe, and the world, with high rates of illness and death, so did symptoms of anxiety, depression, post-traumatic stress disorder, stress, and psychological distress. This study examines the relationship between gender and psychological distress among Latvians over 50 years old within the first 6 months of the COVID-19 pandemic. Data from Wave 8 COVID-19 data of the Survey of Health, Aging, and Retirement in Europe (SHARE) as an early data version of the SHARE Corona survey conducted between June and August 2020. It features the data collected by telephone (CATI) on topics related to COVID-19 for a large sub-sample of SHARE panel respondents. This study examined a sample of 980 adults over 50 years old in Latvia. Bivariate analysis were performed utilizing the Pearson chi-square test for association to examine differences in symptoms of psychological distress by gender during the first six months of the COVID-19 pandemic. Level of significance was determined by the p-value test statistic. Alpha level was established at .05. A chi-square test for association shows that there were statistically relationships between gender and feeling nervous (X2 (2, N = 976) = 22.11, p < .001), feeling depressed (X2 (3, N = 976) = 10.95, p < .01), and trouble sleeping (X2 (2, N = 976) = 20.40, p < .001). This study rejects the null hypothesis that no differences exist between the genders, as women reported greater psychological distress during the first 6 months of the COVID-19 pandemic. Additional multi-method analysis is consistent with these findings and concludes that this is due to the greater concern women report for family, and the burden which placed limitations on meeting their children and grandchildren.

**Key messages:**

- Gender differences exist in experiences of psychological distress during the early COVID-19 pandemic among the aging population in Latvia.
- With the high rates of illness and death associated with the COVID-19 pandemic, so were symptoms of anxiety, depression, post-traumatic stress disorder, stress, and psychological distress.

